# Identification and characterization of lysine-rich proteins and starch biosynthesis genes in the *opaque2* mutant by transcriptional and proteomic analysis

**DOI:** 10.1186/1471-2229-13-60

**Published:** 2013-04-12

**Authors:** Mo Jia, Hao Wu, Kasi L Clay, Rudolf Jung, Brian A Larkins, Bryan C Gibbon

**Affiliations:** 1 Department of Biology, Baylor University, One Bear place #97388, Waco, TX 76798, USA; 2 Pioneer Hi-Bred International, Inc., Johnston, IA 50131, USA; 3 Department of Plant Sciences, University of Arizona, Tucson, AZ 85721, USA

**Keywords:** Opaque endosperm, Opaque2, Quality protein maize, Starch biosynthesis, Protein quality

## Abstract

**Background:**

The *opaque2* mutant is valuable for producing maize varieties with enhanced nutritional value. However, the exact mechanisms by which it improves protein quality and creates a soft endosperm texture are unclear. Given the importance of improving nutritional quality in grain crops, a better understanding of the physiological basis for these traits is necessary.

**Results:**

In this study, we combined transcript profiling and proteomic analysis to better understand which genes and proteins are altered by *opaque2* in the W64A inbred line. These analyses showed that the accumulation of some lysine-rich proteins, such as sorbitol dehydrogenase and glyceraldehyde3-phosphate dehydrogenase, was increased in mature kernels and may contribute substantially to the lysine content of *opaque2* endosperm. Some defense proteins such as beta-glucosidase aggregating factor were strongly down regulated and may be regulated directly by *opaque2*. The mutant also had altered expression of a number of starch biosynthesis genes and this was associated with a more highly crystalline starch.

**Conclusions:**

The results of these studies revealed specific target genes that can be investigated to further improve nutritional quality and agronomic performance of high lysine maize lines, particularly those based on the presence of the *opaque2* mutation. Alteration of amylopectin branching patterns in *opaque2* starch could contribute to generation of the soft, starchy endosperm.

## Background

Maize is a major food and feed crop, and the acreage devoted to maize cultivation is expected to increase significantly over the next several decades due to greater demand for the grain [1]. The majority of the maize crop is used to feed livestock, but in substantial parts of Central America, Africa and Asia, maize is the primary food staple for humans. In order to maximize land productivity, the nutritional quality of crops should be one of the factors considered, along with water and nitrogen use efficiency, yield, pest resistance and other determinants of crop productivity [2].

Maize protein is deficient in the essential amino acids lysine and tryptophan, which limits its value for monogastric animals. Therefore, for the past several decades there have been efforts to create maize lines with increased essential amino acid content. In the 1960s the research groups of Mertz and Nelson at Purdue University identified several mutants with increased lysine content, *opaque2* (*o2*) and *floury2* in particular, had substantially higher essential amino acid content [3,4]. However, these mutations result in a soft, chalky endosperm phenotype that is not suitable for agronomic production because of increased susceptibility to insect and fungal pests and decreased yields [5,6]. The *O2* gene was found to encode a b-zip transcription factor [7] that regulates expression of several genes in the endosperm, notably those encoding the 22 kDa α-zein storage proteins [8]. The substantial reduction in synthesis of α-zeins results in smaller, less numerous protein bodies and a concomitant increase in non-zein endosperm proteins [3]. These changes in protein accumulation result in an endosperm that has nearly twice the lysine and tryptophan content of wild-type maize [3], which substantially improves its value for monogastric animals [9]. Therefore, breeders began recurrent selection of *o2* lines with high lysine and a hard endosperm, called Quality Protein Maize [10].

Recently, considerable progress has been made developing maize lines and optimizing amino acid balance using transgenic [11-13] and conventional breeding approaches through marker-assisted selection [14,15]. The most successful transgenic strategies have been specific knock down of zein storage protein or lysine catabolism gene expression with RNA interference (RNAi) approaches [11-13,16]. Reduced synthesis of the lysine-poor zein proteins and compensatory increases in other proteins dramatically improves the nutritional quality of the grain. The underlying mechanism for rebalancing amino acid content for both *o2* and RNAi is unclear, although it depends on reduced synthesis of the zein storage proteins and a compensatory increase in non-zein protein content [17]. Generally, the total protein content is only slightly depressed relative to wild type kernels [12,17,18], and knocking down 19- and 22-kDa α-zeins in high or low protein lines by RNAi only modestly changes total protein content from the parental levels, suggesting that total protein content is under genetic control [17]. It is possible that competition between mRNA transcripts for ribosomes is responsible for the final protein composition, as has been proposed for soybean [19].

Despite these advances in developing maize lines with higher nutritional value, the underlying physiological and molecular mechanisms that cause soft kernels is still not well understood. Several studies have investigated the changes in transcriptional patterns caused by the *o2* mutation [20-23]. Consistent observations among them point to pleotropic changes in gene expression, but it has been difficult to identify physiological pathways that explain the soft kernel phenotype and changes in protein synthesis that contribute to the improved amino acid composition of the endosperm. Genes related to endoplasmic reticulum (ER) stress responses are consistently up regulated in opaque mutants [22], as are many genes in the glycolytic pathway and others that are typically associated with physiological responses to anoxic stress, such as alcohol dehydrogenase and sorbitol dehydrogenase [21,22,24], but their roles in the expression of the opaque phenotype are not clear. Proteomic analysis of protein accumulation during *o2* development is generally consistent with the pattern of gene expression observed by microarray analysis [25].

In this study we analyzed gene expression in *o2* endosperm using an amplified fragment length polymorphism (AFLP)-based approach that is open-ended and does not depend on known or predicted gene sequences. We also performed a proteomic analysis of mature seeds to identify specific proteins that contribute disproportionately to the increased lysine and tryptophan content in order to relate these more abundant gene products to gene expression in maturing endosperm. In addition to confirming overall gene expression patterns previously described for *o2* mutants, we identified a number of other differences in mRNA transcript levels compared to wild type endosperms. Several gene products related to defense responses were also substantially down regulated in *o2* endosperm, which could further explain its greater susceptibility to ear rots and insect pests. Expression of starch biosynthetic genes was altered in *o2* and was associated with changes in starch granule structure. Furthermore, analysis of protein accumulation in mature seeds revealed a few lysine-rich proteins that were substantially more abundant in *o2* endosperm. These changes could explain a significant fraction of the increased lysine content in W64A*o2*. How changes in gene expression, protein content and starch structure contribute to the development of opaque endosperm is discussed.

## Results and discussion

### Overview of transcript profiling

**Figure 1:**
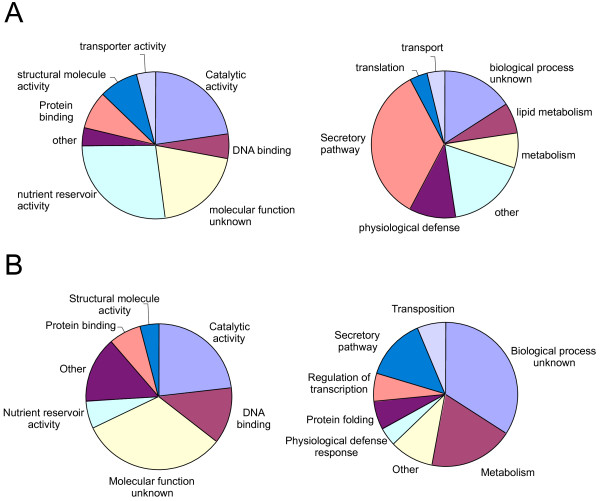
**GO classification for genes with altered expression in *o2*.** Genes were assigned to GO molecular function (**A**) and biological process (**B**). The plots on the left are genes down regulated in *o2* and the plots on the right are genes up regulated in *o2*.

To systematically compare gene expression patterns between W64A + and W64A*o2* at the most metabolically active stage of endosperm development*,* transcript profiling was performed at Curagen Corp. (New Haven, CT) by GeneCalling™ [26] at 22 days after pollination (DAP). The GeneCalling™ approach does not rely on a priori knowledge of gene sequences and can therefore identify expression differences for genes that are not present in sequence databases. cDNA fragments were generated with 47 different pairs of restriction enzymes, and the expression levels of the corresponding gene fragments were compared. A total of 470 putative genes were identified as differentially expressed in W64A + and W64A*o2* by the GeneCalling™ software using a t-test. The sequence of a subset of the differentially expressed gene fragments was confirmed by oligonucleotide competition, “poisoning”, with an unlabeled gene-specific primer or by cloning and sequencing the fragments if poisoning failed. The identities of 274 gene fragments ranging from 50 to 500 bp were confirmed and represented a total of 151 gene products. Further characterization of these genes was obtained by BLASTN and BLASTX analyses against Genbank and Maize Genome Sequence databases (http://www.maizesequence.org). The molecular functions and biological processes were annotated using the gene ontology database (G.O.; http://www.geneontology.org) and classification of their molecular functions and biological processes are illustrated in Figure 1. A comprehensive table of differentially expressed genes and their properties is provided in Additional file 1: Table S1. Twenty-six distinct biological functions were affected in W64A*o2*, including carbohydrate metabolism and stress responses, which are associated with the altered endosperm phenotype of the *o2* mutant. Specifically, 70 genes corresponding to 23 functional groups were up regulated and 81 gene fragments belonging to 16 groups were down regulated in *o2*.

### GO functional classes of up regulated and down regulated genes

As expected, a large number of down regulated genes have a molecular function associated with nutrient reservoir activity (Figure 1), which is due to the accumulation of several 19- and 22-kD α-zein genes and the 27-kD γ-zein being significantly reduced in *o2*. These proteins are encoded by large gene families with highly conserved sequences and are well-characterized targets of O2 regulation [27,28]. Reduction in nutrient reservoir gene function in *o2* is followed by catalytic activity (23%), structural molecule activity (8%), protein binding (8%), DNA binding (6%), transporter activity (4%) and other minor categories (4%), such as ion binding and enzyme regulator activity. Among biological processes, 34% of the down regulated genes participate in the secretory pathway, which may reflect a response to the reduced accumulation of the ER-resident zein storage proteins. Other down regulated functional categories include physiological defense (11%), metabolism (8%), lipid metabolism (7%), transport (4%), translation (4%) and other (17%). There are 20% and 16% of the genes with unknown molecular function and biological process, respectively.

For genes that are up regulated in *o2*, only a small proportion have the function of nutrient reservoir activity (6%). Instead, the largest proportion of the up regulated genes have catalytic activities (23%), followed by DNA binding (13%), protein binding (7%), structural molecule activity (4%) and other (15%). This is consistent with metabolism being the most affected biological function (19%), followed by secretion (14%), protein folding (6%), transcription (6%), transposition (6%), and physiological defense response (4%). Of the up regulated genes, 32% could not be assigned a molecular function and 34% could not be assigned to a biological process.

### Genes down regulated in o2

As expected, members of the zein gene family were significantly down regulated in *o2* (19 kDa and 22 kDa α-zeins and 27 kDa γ-zein). There were a few zein genes with increased expression in *o2*. However, this method of analyzing transcripts is very sensitive to allelic differences, and the up regulated zein genes may represent such alleles. Several genes that are reported to participate in defense responses to biotic and abiotic stresses were also significantly down regulated in *o2,* such as a ribosome-inactivating protein (RIP) b-32, which has a defensive role against pathogens and viruses and a well-known target of O2 regulation in maize [29,30]. A beta-glucosidase aggregating factor-like protein (BGAF) was also strongly down regulated; such proteins are reported to be involved in defense against pathogens and herbivores [31,32]. The BGAF-like protein may be a particularly interesting gene to study further because it has an O2 consensus binding sequence [24,33] at −227 nt from the predicted transcription start. However, there were several other defense-related transcripts that were down regulated to a lesser extent, subtilisin-chymotrypsin inhibitor CI-1B (CI-1B), which responds to wounding [34], flower-specific gamma-thionin (defensin SD2), which is toxic to animal cells and defends against parasites [35], and basal layer antifungal protein2 (BAP2). It is possible that the high sensitivity of *o2* to fungal and insect pests is due to the synergistic effect of reducing both b-32 and BGAF protein levels in *o2* endosperm.

Several ribosomal proteins, such as the 40S subunit protein S3a and the 60S ribosomal subunit protein L19-3, and the 18S RNA gene, the structural RNA for the small subunit of eukaryotic cytoplasmic ribosomes, were all down regulated in *o2*. Some of these changes in ribosomal constituents may be cellular responses to the changes in the overall mRNA pool, which lacks the abundant ER-targeted α-zein mRNAs in *o2*. Other down regulated transcripts included NAC (NAM, ATAF, and CUC transcription factor) domain-containing protein 48, which is predicted to function as a plant specific transcription factor involved in a variety of developmental events, as well as in biotic and abiotic stress responses [36]. Genes that function in signal transduction, such as YT521-B-like family protein, glutathione S-transferase GST 31, protein FAR-RED IMPAIRED RESPONSE 1, also showed decreased expression in *o2*. The role of these transcription factors and signal transduction proteins have in the formation of the opaque phenotype, if any, is unclear.

Several genes that function in amino acid metabolism were also down regulated, including tryptophan aminotransferase (TA1) and ketol-acid reductoisomerase which catalyzes two steps of the biosynthetic pathway of the branched-chain amino acids valine, leucine and isoleucine [37] and alanine-glyoxylate aminotransferase 2. Surprisingly, LKR-SDH1 is thought to be regulated by O2 [38], yet the transcript expression was not significantly different between W64A + and W64A*o2* and this may indicate that its expression is influenced by genetic background or environment. It may be the case that a large number of amino acid biosynthetic enzymes are regulated to some extent by O2. The yeast homolog of O2, the b-zip transcription factor GCN4 (General Control Non-derepressible 4), is known to induce the expression of a large number of amino acid biosynthetic genes in response to amino acid starvation [39] and *gcn4* mutants can be complemented by expression of the maize *O2* gene [40].

Several genes related to cell structure and development were down regulated in W64A*o2*, including: katanin p60 ATPase which is involved in the regulation of microtubule dynamics [41] and regulates plant cell division and growth [42]; arabinogalactan protein (AGP), which serves as a marker of cellular identity and fate, and functions in plant vegetative growth and development as well as secondary cell wall thickening and programmed cell death [43]; brassinosteroid biosynthesis-like proteins, which are natural growth regulators required for post-embryonic growth [44]; and maternal effect embryo arrest 21 (MEE21), which regulates embryo development and maturation [45]. Although it is not known how such proteins influence the opaque phenotype, it is possible that they could cause changes in cellular organization that predispose the endosperm cells to develop the characteristic gaps between starch granules that is a hallmark of opaque endosperm.

### Genes up regulated in o2

A number of genes encoding primary carbohydrate metabolism enzymes were up regulated in W64A*o2*. Two enzymes of the glycolytic pathway were up regulated, cytosolic triosephosphate isomerase (TIM) and cytosolic phosphoglycerate kinase (PGK). Fructokinase-1, which functions at the entry point into glycolysis via the formation of glucose-6-phosphate and maintains the flux of carbon towards starch formation, was increased 1.85-fold. Many of these changes in glycolytic enzyme expression and the up regulation of alcohol dehydrogenase 1 by 2.86-fold were consistent with a hypoxic response. It has been shown that the maize endosperm is a highly anoxic environment compared to the embryo, and that this is likely to result in the shunting of carbon into starch rather than oil [46]. However, it is not clear why the *o2* mutant would display increased hypoxic responses, and the proportion of starch in *o2* endosperm is essentially identical to wild type in the W64A background (not shown).

Enzymes involved in starch biosynthesis were increased in *o2*, including granule-bound starch synthase I (GBSSI), which is required for the synthesis of amylose. Enzymes required for amylopectin synthesis were also up regulated, including pullulanase-type starch debranching enzyme1 (Zpu1), which hydrolyzes the α-1,6-glucosic linkages of polyglucans, 1,4-alpha-glucan-branching enzyme 2 (BE2), which catalyzes the formation of α-1,6 glucan and is required for amylopectin synthesis at the surface of the starch granule. Trehalose-6-phosphate synthase was also increased, which has been implicated in the redox activation of ADP-Glc phosphorylase, the enzyme that catalyzes the first committed step of starch synthesis [47,48]. Prior work has shown that the biochemical properties of starch are altered in opaque mutants [49], but the underlying mechanism is still not clear. The change in expression of one or more starch biosynthesis enzymes could result in the observed properties of *o2* starch, although altering the expression or mutation of one starch biosynthetic enzyme can have complex effects on multiple enzyme activities.

Several proteins involved in the maintenance and folding of proteins in the ER were up regulated. The expression of the calcium-dependent protein chaperones, Calnexin, calreticulin2 and the chaperone DNA J2, were increased approximately two-fold in W64A*o2.* The small cytoplasmic chaperones, 16.9 kDa class I heat shock protein 3 and heat shock protein18c were also up regulated. Other ER enzymes involved in the oxidation of cysteine to form disulfide bonds including protein disulfide isomerase (PDI) and ER Membrane-Localized Oxidoreductase 1 (ERO1) were increased [50]. These genes are related to the unfolded protein response and their up regulation is likely due to alteration of protein body structure in the ER [22].

Stress-response and defense genes up regulated in W64A*o2* included the following: alliin lyase 2 (alliinase) and cystatin 6, which are part of the defense response against herbivores [51,52]; xylanase inhibitor protein 1 and glycine–aspartic acid–serine-leucine (GDSL)-motif lipase/hydrolase-like protein, both of which are involved in the defense against fungal pathogens [53,54]; and a Pi starvation-induced protein and an ABA-responsive 40 kDa protein [55-57]. A MAP kinase was up regulated, as were several MAP kinase responsive genes. These include the respiratory burst oxidase protein, homolog B (RBOHB), and an inducible form of the NADPH oxidase, a downstream effector in the mitogen-activated protein kinase (MAPK) regulated signaling pathway that generates reactive oxygen species (ROS) and triggers innate immunity in response to various stresses [58]*.* Additionally, the WRKY transcription factor was up regulated, which is phosphorylated and activated by MAPKs in response to biotic and abiotic stresses [59]. These up regulated stress responses are unlikely to confer enhanced resistance to pests and most likely represent pleiotropic responses to mutation of *o2*, because there is ample evidence that *o2* is much more susceptible to pests.

### Proteomic comparison of opaque2 and wild type lines

**Figure 2:**
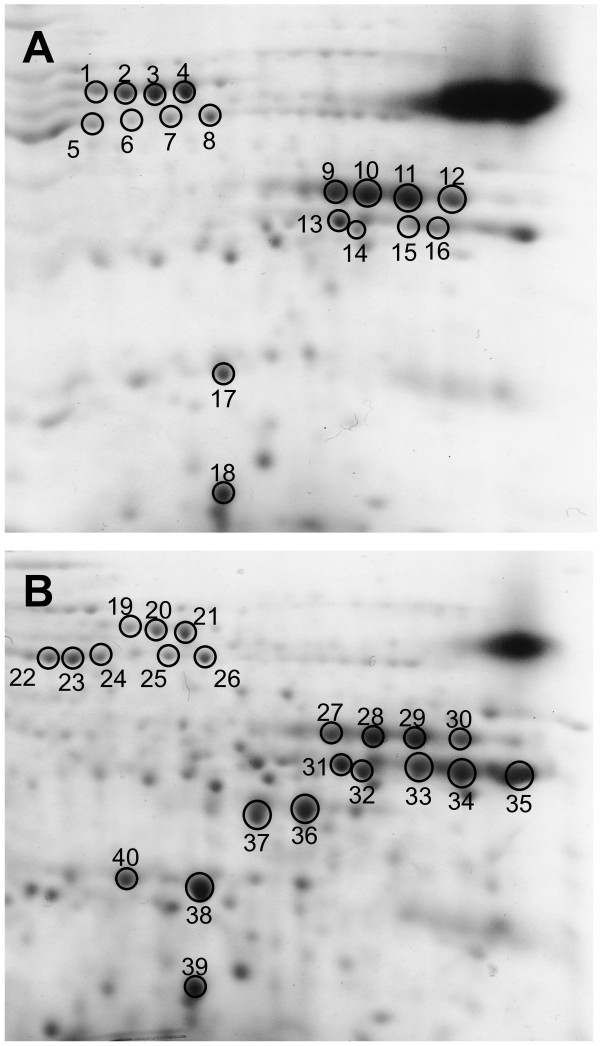
**2D SDS-PAGE analysis of W64A + and W64A*o2*.** Non-zein proteins from W64A + (**A**) and W64A*o2* (**B**) lines were extracted from mature endosperm flour and separated by 2D SDS-PAGE. Circled, numbered spots were excised from gels and protein identities were determined by MALDI-TOF peptide mass mapping and correspond to the rows in Table 1.

In order to detect differences in non-zein protein accumulation in W64A*o2* and wild type lines, we performed 2D SDS-PAGE analysis with equal amounts of non-zein proteins purified from mature endosperms using a borate extraction method [60]. Mature kernels were analyzed in order to determine if abundant non-zein proteins that contribute to increased lysine were consistent with their gene expression during endosperm development. After visualization and alignment of gels, 40 protein spots that were differentially resolved or showed altered accumulation levels were excised from gels for identification (Figure 2). Proteins of interest were identified by MALDI-TOF peptide mass mapping of trypsin digests of the protein spots. GBSSI, enolase 1, legumin-like protein, GAPDH, TIM and SDH showed increased accumulation in *o2*, while enolase 2 and HSP3 showed no alteration in accumulation (Table 1). Many of the largest differences in protein accumulation were reflected in the transcript levels measured by transcript profiling at 22 DAP. The exceptions were GBSSI and enolase 1, which had inconsistent fold-changes in multiple studies [20-22]. This could be due to differences in genetic backgrounds of the lines analyzed or the different environments in which the materials were grown. However, in the W64A background the transcript profiling and proteomic data showed enolase 1 accumulation was higher in *o2* (Figure 2). Notably, there was a significant increase (~1.8-fold) in the accumulation of GAPDH, which contains over 8% lysine, but was not found to be significantly different in transcript abundance in the transcript profiling data. Likewise there was a >2.5-fold increase in some SDH1 isoforms and this protein contains 4% lysine, which is lower than GAPDH, but nearly two-fold higher than the typical total lysine content of wild type maize endosperm. The increase in GAPDH and SDH1 could contribute significantly to the elevated level of lysine in W64A*o2*, and the expression of these very abundant proteins may be associated with the expression of translation elongation factor 1A (EFIA), which is correlated with lysine content in maize endosperm but is not sufficient to explain the total increase in lysine content [61].

**Table 1:** Identity and expression differences of proteins separated by 2D SDS-page

**Spot**	**Genbank**	**Annotation**	**Fold-change**
1	P04713	Granule-bound starch synthase 1	
2	P04713	Granule-bound starch synthase 1	
3	P04713	Granule-bound starch synthase 1	
4	P04713	Granule-bound starch synthase 1	
5	NP_001105896	enolase 1	
6	NP_001105896	enolase 1	
7	NP_001105371	enolase 2	
8	NP_001105371	enolase 2	
9	BAB11045	sorbitol dehydrogenase-like protein	
10	BAB11045	sorbitol dehydrogenase-like protein	
11	BAB11045	sorbitol dehydrogenase-like protein	
12	BAB11045	sorbitol dehydrogenase-like protein	
13	ACG32147	eukaryotic translation initiation factor 2 alpha	
14	AAO63267	Legumin-like protein, complete	
15	Q43247	Glyceraldehyde-3-phosphate dehydrogenase	
16	Q43247	Glyceraldehyde-3-phosphate dehydrogenase	
17	NP_001140424	triosephosphate isomerase	
18	ACG35098	17.4 kDa class I heat shock protein 3	
19	P04713	Granule-bound starch synthase 1	**−2.5**^**1**^
20	P04713	Granule-bound starch synthase 1	**−2.1**
21	P04713	Granule-bound starch synthase 1	−1.5
22	NP_001105896	enolase 1	1.3
23	NP_001105896	enolase 1	1.2
24	NP_001105896	enolase 1	1.1
25	NP_001105371	enolase 2	1.1
26	NP_001105371	enolase 2	1.3
27	BAB11045	sorbitol dehydrogenase-like protein	−1.5
28	BAB11045	sorbitol dehydrogenase-like protein	−1.2
29	BAB11045	sorbitol dehydrogenase-like protein	**−1.4**
30	BAB11045	sorbitol dehydrogenase-like protein	−1.2
31	ACG32147	eukaryotic translation initiation factor 2 alpha	1.2
32	AAO63267	Legumin-like protein, complete	1.5
33	Q43247	Glyceraldehyde-3-phosphate dehydrogenase	**1.7**
34	Q43247	Glyceraldehyde-3-phosphate dehydrogenase	**1.8**
35	AAA87580	Glyceroldehyde-3-phosphate dehydrogenase	1.6
36	NP_001149440	sorbitol dehydrogenase homolog1	**2.7**
37	ABA70761	sorbitol dehydrogenase	**2.9**
38	NP_001140424	triosephosphate isomerase, cytosolic	1.5
39	ACG35098	17.4 kDa class I heat shock protein 3	1.2
40	NP_001140424	triosephosphate isomerase, cytosolic	1.6

^1^Bold values indicate significant difference by ANOVA (p < 0.05; n = 3).

### Validation of gene expression

Quantitative real-time polymerase chain reaction (qRT-PCR) was performed for several genes encoding both up and down regulated transcripts in W64A*o2* to validate the results from the transcript profiling experiment with endosperms from both genotypes at 22 DAP. Retinoblastoma-related protein 1 (RRB1) was used as the reference gene, because it is consistently expressed in both genotypes (see Materials and Methods). The RIP gene, b-32, was chosen as a positive control for qRT-PCR, because it is known to be down regulated in *o2* mutants [30]. Genes were selected based on the following categories: 1) genes with expression that was highly reduced in the *o2* mutant in the profiling experiment, such as proteosome regulatory subunit AAA-ATPase (AAA-ATPase), stem-specific protein (TSJT1), 16-kDa oleosin, CI-1B and BGAF, which had not been characterized in previous studies; 2) starch biosynthesis genes, such as Zpu1, starch branching enzyme IIb (BEIIb) and GBSSI; 3) genes related to carbohydrate metabolism that were changed in the transcript profiling or 2D SDS-PAGE analysis, including GAPDH, sorbitol dehydrogenase 1 (SDH1), TIM, enolase 1 and PGK; and 4) other genes that showed changes in W64A*o2* according to profiling results, such as actin2, legumin1, 17.4 kDa class I heat shock protein 3 (HSP3) and LKR-SDH1.

**Figure 3:**
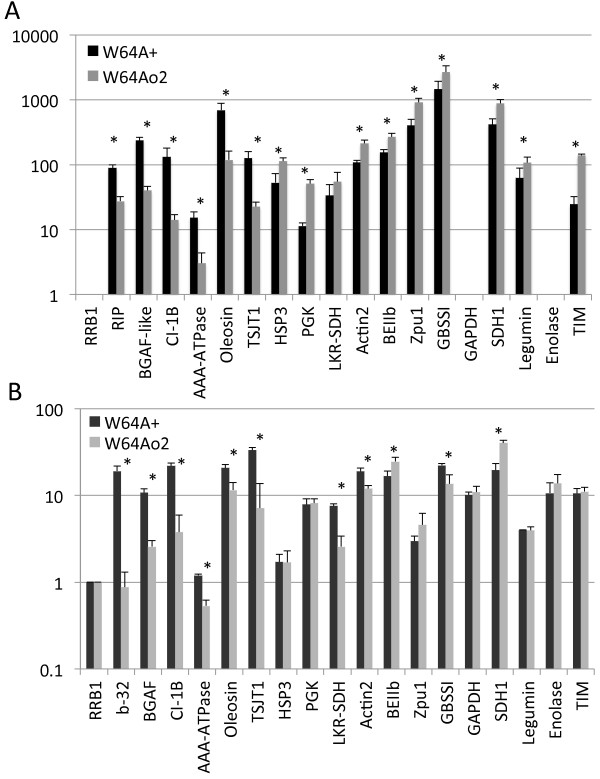
**Confirmation of genes or proteins altered in W64A*o2 *by qRT-PCR.** Expression of the indicated genes was analyzed in 22 DAP endosperms of W64A + and W64A*o2* by GeneCalling transcript profiling (**A**) or qRT-PCR to confirm the difference in expression in the transcript profiling, or to measure the expression levels of genes that were differentially expressed by 2D SDS-PAGE analysis (**B**). All expression values are normalized relative to the expression of RRB1. Asterisks indicate significantly different expression using the two-tailed t-test at a level of p < 0.05. Note that the Y-axis is logarithmic to accommodate the wide differences in gene expression levels among the transcripts. Missing columns in A indicate that the gene was not among the genes that had a confirmed identity in the transcript profiling data.

The relative expression levels of transcripts among various samples in the qRT-PCR generally agreed with the profiling results (Pearson correlation coefficient *r* = 0.80, ANOVA p < 0.001; Additional file 2: Figure S1). However, in some cases the transcript fold-changes measured by Gene Calling were higher than those from qRT-PCR. The transcript level of the known O2-regulated gene b-32 was significantly reduced in *o2* by both transcript profiling and qRT-PCR (Figures 3A and 3B)*,* consistent with previous reports [20-22]. The transcript levels of BGAF, 16 kDa oleosin, CI-1B, TSJT1 and AAA-ATPase were all significantly reduced in *o2,* compared to W64A + by qRT-PCR (Figure 3B) and Gene Calling (Figure 3A and Additional file 1: Table S1). qRT-PCR analysis also confirmed the expression of starch synthesis genes that were found to be up regulated in *o2* such as Zpu1 and BEIIb [20,22] (Figure 3). Although in the case of Zpu1, the increase was greater in the GeneCalling results (2.27-fold versus 1.55-fold in qRT-PCR). These results indicate that the majority of changes in gene expression are the result of the *o2* mutation, and not the genetic background or environmental conditions in the field. On the other hand, the expression of some genes was not consistent, notably LKR-SDH and GBSSI. As stated previously LKR-SDH was not significantly different in the transcript profiling data (Figure 3A), yet when measured by qRT-PCR in individuals grown in a different environment there was a significant difference (Figure 3B). Likewise, the difference in the transcript level of GBSSI was not consistent among previous reports [20-22]. GBSSI transcript increased in *o2* according to the GeneCalling analysis, but decreased significantly based on the qRT-PCR analysis. These results together with data from prior studies indicate that expression of GBSSI and LKR-SDH may be dependent on both genotype and environmental conditions.

Carbohydrate metabolism-related genes significantly affected in *o2* by either GeneCalling or 2D SDS-PAGE analysis were also examined by qRT-PCR. Of the genes that were tested, only SDH1 showed significantly higher expression in *o2* at 22 DAP (Figure 3). Finally, the expression of HSP3 and actin2 measured by qRT-PCR did not agree with the GeneCalling results, but the decreased level of actin2 in *o2* was observed in other experiments [21,22]. There is a possibility that this difference was due to primer specificity, since both HSP and actin belong to multigene families and there are other family members that share significant sequence similarity. Therefore, multiple gene family members could be detected at the same time in qRT-PCR.

### Western blot analysis of opaque2 and wild type lines

**Figure 4:**
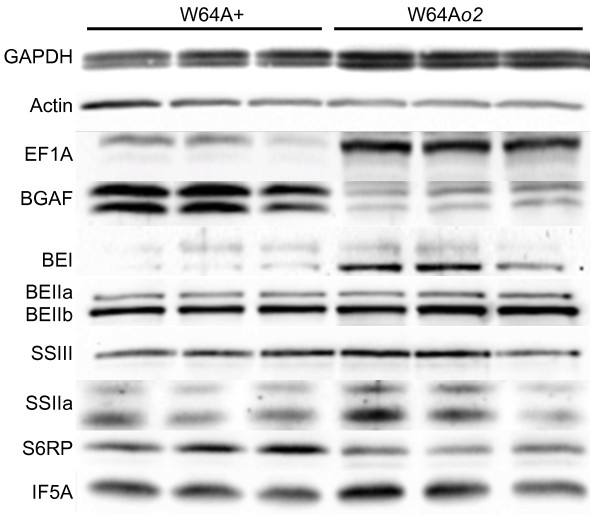
**Western blot analysis of selected proteins in W64A + and W64A*o2 *.** Western blots were performed using antisera against the proteins indicated on the left. Three replicate samples for each line were obtained from independent ears frozen at 22 DAP and 25 μg of protein from each was separated by SDS-PAGE followed by western blotting. Each band was analyzed by densitometry and the fold change values calculated for each protein and the values are presented in Table 2.

Western blot analysis of 22 DAP W64A + and W64A*o2* maize endosperm was performed to extend the transcript profiling and proteomic analysis (Figure 4); quantitative measurement by densitometry is shown in Table 2. In contrast to the gene expression data, the *o2* mutants showed an increase in GAPDH protein abundance by both 2D SDS-PAGE and by western blot analysis. This suggests that the GAPDH protein may be particularly stable in endosperm cells and therefore accumulates to a substantially higher level than indicated by its transcript abundance during seed development. Although expression of actin2 was increased in *o2* in the transcript profiling data, no measurable protein difference was observed on western blots (Figure 4). However, the anti-actin antibody available is reported to recognize many isoforms of the protein across multiple kingdoms. Therefore it was not specific for the product of the gene that was up regulated in the present analysis. As expected, EF1A was significantly higher in *o2*, whereas other translation-related factors were either slightly higher (translation initiation factor 5A, IF5A) or slightly lower (ribosomal protein S6, S6RP) in W64A*o2*. However, there were no measurable differences in eukaryotic translation initiation factor 4G (eIF4G), eukaryotic translation initiation factor 2 alpha subunit (eIF2α), or eukaryotic translation initiation factor 4E (eIF4E) (not shown). Analysis of starch biosynthetic enzymes showed that BEIIa and BEIIb were not different between *o2* and wild type. However, there was increased accumulation of starch synthase IIa (SSIIa) and starch branching enzyme I (BEI) in *o2*. Both of these enzymes have significant effects on starch structure when mutated or knocked down by RNAi, which results in the accumulation of amylopectin with relatively short glucan chains [62,63]. In contrast, BEI preferentially produced longer chain length branches (>16) compared with BEIIb, which preferentially produced shorter branches (<12) in an *in vitro* assay [64]. Together, these observations suggest that in the W64A*o2* mutant the average chain length of amylopectin branches would be greater than in W64A + .

**Table 2:** **Densitometry analysis of western blots of wild type and *opaque2 *** **endosperm extracts**

	**W64A+**		**W64A** ***o2***			
**Protein**	**Mean**	**SD**	**Mean**	**SD**	**Fold-change**	**p**^**1**^
GAPDH	9.80	0.65	14.73	0.94	1.50	**0.002**
EF1A	2.57	0.82	7.19	0.62	2.79	**0.001**
S6RP	7.16	0.59	5.03	0.91	−1.43	**0.03**
IF5A	8.19	0.88	9.37	1.61	1.14	0.33
Actin	2.84	0.78	2.47	0.23	−1.15	0.47
BGAF	7.55	1.62	2.23	0.38	−3.38	**0.005**
SSIIa	2.68	0.43	3.74	0.87	1.40	0.13
SSIII	5.22	0.79	6.35	0.84	1.22	0.17
BEI	0.69	0.07	2.04	0.54	2.97	**0.01**
BEIIa	3.08	0.17	3.74	0.67	1.21	0.18
BEIIb	5.83	0.64	7.52	1.02	1.29	0.07

^1^p-value for two tailed Student’s t-test. Bold figures are significantly different at a level of p < 0.05 (n = 3).

### Analysis of starch

**Figure 5:**
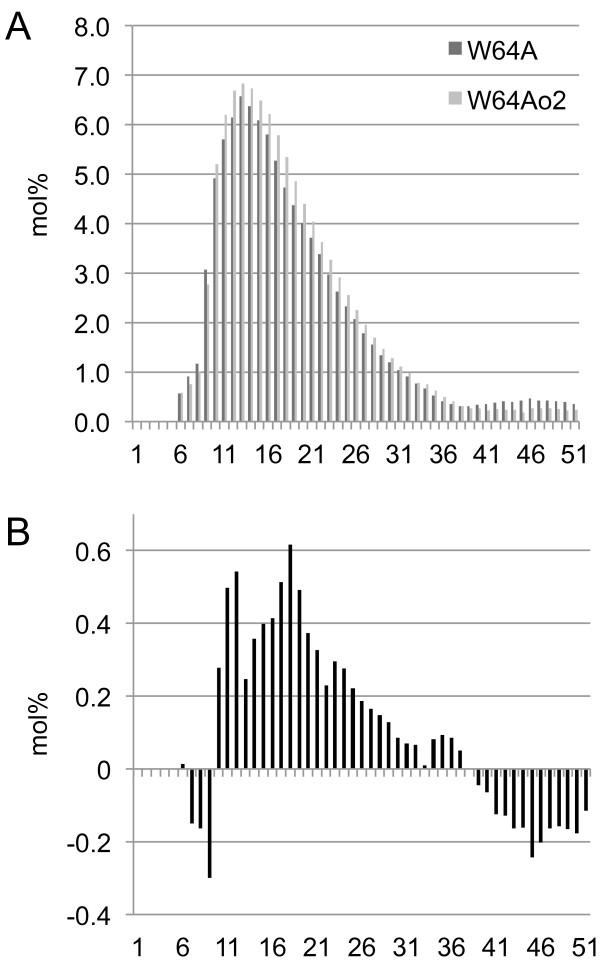
**Amylopectin branch length analysis.** Solubilized starch was de-branched in the presence of isoamylase and the resulting glucan chains were separated by capillary electrophoresis. (**A**) Histograms of the distribution of glucan chains were similar but W64A*o2* was shifted toward a higher degree of polymerization. (**B**) Difference plot was calculated by subtracting the W64A + values from the W64A*o2* values showed a substantial increase in chains with a degree of polymerization between 15 and 25 glucose subunits. The histograms represent the average of three replicates for each genotype.

The expression of several starch biosynthesis genes varied between W64A + and W64A*o2* based on gene expression analysis and 2D SDS-PAGE. Interestingly, *o2* was the only mutant among eight different isogenic opaque mutant lines that showed significant expression differences in starch biosynthesis genes ([22], Gibbon and Larkins unpublished). Because levels of several starch biosynthesis enzymes were altered in *o2*, SSIIa and BEI in particular, the properties of the starch from W64A + and W64A*o2* were analyzed by differential scanning calorimetry (DSC) to determine if these changes affected the starch structure. The onset and peak endotherm temperatures as well as the total enthalpy of gelatinization were significantly higher for W64A*o2* (Table 3). The higher values for these thermal properties in *o2* are consistent with starch that has longer amylopectin branches and higher crystalline starch content. To further characterize the structure of the starch, the amylopectin branch length distributions of W64A + and W64A*o2* were measured. Debranched starch glucans were separated by capillary electrophoresis and the resulting branch length distributions were compared (Figure 5). The two genotypes had similar molar percent content of glucans, but the distribution of glucans from W64A*o2* was shifted toward a higher degree of polymerization (Figure 5A). A difference plot clearly showed a marked increase in glucan chains with a degree of polymerization between 15 and 25 glucose subunits in W64A*o2* (Figure 5B). These results were similar to what was previously observed for *o2* in the CM105 inbred line [49]. Together, the western blot analysis and analysis of starch structure suggest that enhanced BEI or SSIIa activity results in amylopectin with significantly longer glucan chains in W64A*o2*. These changes in the crystallinity and branching pattern of W64A*o2* starch may alter the association of the starch granules with endosperm proteins and thus promote formation of a soft, opaque phenotype.

**Table 3:** **DSC analysis of W64A + and W64A** ***o2 *** **starch**

	**W64A+**	**W64A** ***o2***	**p**^**1**^
Onset (°C)	68.02 ± 0.659	71.38 ± 0.169	<.0001
Peak Endotherm (°C)	71.84 ± 0.846	74.65 ± 0.172	<.0001
Total Enthalpy (J/G)	11.29 ± 0.83	14.39 ± 1.00	<.0001

^1^Student’s t-test.

## Conclusions

The analysis of *opaque2* transcription patterns by Gene Calling significantly expanded the results of previous studies using microarrays, and by combining transcript profiling with proteomic analysis, we were able to document the presence of certain abundant lysine-containing proteins related to primary carbon metabolism. This is consistent with prior proteomic analyses of developing kernels [25], but the relative levels appear to be proportionally much higher in mature kernels. The two proteins that appeared to be most abundant were SDH1 and GAPDH, which have lysine contents of 4.2 and 8.5 percent, respectively. Especially for GAPDH, its relatively high accumulation in mature endosperm could contribute a substantial proportion of the total increased lysine observed in *o2*. The results could explain the relatively high lysine content of W64A*o2* endosperm, and the basis of the phenotypic variability for this trait among maize inbreds [65].

Additional genes that contribute to the deleterious phenotypes of *o2* and that appear to be related to pest resistance were identified in this analysis. RIP is a well-known *o2* target gene and plays a role in the defense against fungal pathogenesis [66]. Likewise, BGAF was strongly down regulated, and it is suggested to have a role of concentrating beta-glucosidase at wound sites to promote activation of glycosylated defense compounds [31]. Other down regulated defense proteins included Cl-1B, BAP2, and defensin SD2. Down regulation of such defense proteins may synergistically contribute to the high susceptibility of *o2* to fungal and insect pests. Investigation of these genes in *o2* or modified *o2* backgrounds may aid in the development of better performing high lysine maize lines.

Finally, *o2* was the only opaque mutant to show significant alteration of starch biosynthetic gene expression. In particular, the up regulation of BEI and/or SSIIa appears to explain the production of starch granules that are more highly crystalline in character, which could contribute to the opaque phenotype. Former studies indicated that an alteration in starch granule structure could be an important contributor to the restoration of vitreous endosperm by *o2* modifiers in QPM [49]. Our recent studies indicate that pullulanase activity is significantly higher in QPM and correlates well with the extent of endosperm modification, and this change is most likely due to a reduction in glucan chain length relative to soft *o2* mutants (Wu and Gibbon unpublished data). Therefore, manipulation of starch quality by transgenic means or naturally occurring alleles of BEI or SSIIa may be a way to enhance kernel quality and suppress the opaque phenotype for the improvement of QPM or other high lysine maize lines.

## Methods

### Transcript profiling by GeneCalling™

Plants of the nearly-isogenic maize (*Zea mays* L.) inbred lines W64A + and W64A*o2*[22] were grown in the summer of 1998 in field plots at the Pioneer Hi-Bred International genetic nursery in Johnston, IA. Well-filled ears of each inbred line were harvested 22 DAP and immediately frozen in liquid nitrogen. To minimize the effect of biological variation between ears on the gene expression analysis, equal numbers of endosperms from the middle portion of three ears were pooled. Total RNA was isolated using the PURE*script* kit (Gentra Systems, Inc., Minneapolis), and mRNA profiling was performed at Curagen (New Haven, CT) by GeneCalling™ [26]. In brief, cDNA was synthesized from three independently pooled W64A + and three independently pooled W64A*o2* endosperm samples (biological repetitions). Each of the six cDNA preparations was divided into three aliquots (technical repetitions) to provide nine repetitions per genotype for profiling analysis. Each cDNA aliquot was digested with 47 different combinations of restriction enzyme pairs. Fragments from each digest were ligated to adapters; the fragments were amplified with primers that have unique tags (biotin on one end, fluorescent marker at the other). Labeled fragments were purified using streptavidin beads and resolved by high-resolution gel electrophoresis to generate traces showing peaks whose position and height represented *M*_r_ and abundance of cDNA fragment(s), respectively. GeneCalling™ software compiled a list of differentially abundant fragments and assigned a ranking (significance) to each detected difference. The software further searched a nucleic acid database for fragments with the same length and end sequences and predicted likely gene candidates. The identity of predicted fragments was confirmed by competitive amplification with an unlabeled gene-specific primer (“poisoning”) or by cloning and sequencing the fragment [26]. A file containing the confirmed gene sequence tags is provided as Additional file 3.

### Confirmation of expression differences by quantitative real-time PCR

#### Plant materials

W64A + and *o2* kernels for quantitative polymerase chain reaction (qRT-PCR) and western blotting were grown in Elm Mott, TX during the summer of 2012. The kernels were harvested at 22 DAP and kept frozen at −80°C. Three ears of each genotype were used as three biological replicates. Six endosperms of each ear were dissected and ground to a fine powder in liquid nitrogen using a mortar and pestle. For RNA isolation, up to 0.1 g of the materials were used. For protein extraction, 50 mg were weighed and homogenized in borate extraction buffer (12.5 mM NaBO_3_, 1% [w/v] sodium dodecyl sulfate and 2% [v/v] 2-mercaptoethanol).

#### RNA isolation, cDNA synthesis and qRT-PCR

Total RNA was isolated from frozen endosperms using Purelink™ Plant RNA Reagent (Invitrogen, Carlsbad, CA) following the manufacturer’s instructions. RNA samples were diluted to a final concentration of ~100 ng/μl and quantified on a NanoDrop ND-1000 UV/Vis spectrophotometer (NanoDrop Technologies, Wilmington, DE), the purity of which was checked by the ratio of absorptions at 260 nm and 280 nm and all the samples had a ratio ≥ 2.0. First-strand cDNA was synthesized from 1 μg of RNA using qScript™ cDNA SuperMix (Quanta Biosciences, Gaithersburg, MD) and subsequently diluted 10-fold in water.

Primers for qRT-PCR were designed to amplify a 150–300 bp region of selected genes based on Primer3 Plus software (http://www.bioinformatics.nl/cgi-bin/primer3plus/primer3plus.cgi). Primers were designed for a 62°C annealing temperature and to span exon-exon junctions in order to control for genomic DNA contamination (Additional file 4: Table S2).

For gene expression analysis, qRT-PCR was performed in a 72-well rotor using the Corbett Rotor-Gene™ 3000 (Qiagen, Velancia, CA). Each 20 μl reaction contained 10 μl PerfeCTa® SYBR® Green FastMix® (Quanta Biosciences, Gaithersburg, MD), 2.5 μl 10-fold diluted cDNA or 1 μl plasmid standards with copy numbers from 10^5^ to 10^8^, and 1 μM of each primer. The PCR program was as follows: 50°C hold for 2 min for auto gain optimization, 95°C initial denaturing for 10 min, 50 cycles of 95°C for 15 s and 60°C for 1 min. Melting curves were obtained by heating from 55°C to 95°C with a 1°C per second ramp rate to confirm single amplicons. Expression levels of genes in W64A + and W64A*o2* were normalized against the expression of RRB1 gene in the corresponding genotypes [67], since it was not differentially expressed between the two genotypes in preliminary experiments. Normalization of Gene expression was performed using the Q-Gene Core Module file [68]. Statistical differences of gene expression levels between W64A + and W64A*o2* were evaluated with unpaired two-tailed student’s t-test, and the agreement of gene expression levels from transcript profiling and qRT-PCR results were calculated with Pearson correlation coefficient with significance determined by ANOVA, using the JMP statistical software (SAS Institute Inc., Cary, NC).

### Kernel protein extraction, SDS-PAGE and western blotting

Total soluble proteins from maize kernels were extracted with borate extraction buffer containing 12.5 mM sodium borate, 1% (w/v) SDS, 2% β-mercaptoethanol, pH 10 [69]. One ml of borate extraction buffer was added to 50 mg ground kernels and incubated with shaking for at least 2 h at room temperature. Insoluble cell debris was removed from the crude extract by centrifugation for 15 min at 16,000 × g at room temperature. The cleared protein extracts were aliquoted and stored at −80°C.

Twenty-five μg of total protein from each sample were separated by 12% SDS-PAGE in 1X SDS-PAGE running buffer (25 mM Tris, 192 mM glycine, 0.1% (w/v) SDS) and then transferred to a BioTrace™ PVDF membrane (Pall Corporation, Pensacola, FL) using a TE 22 Mighty Small Transphor Tank Transfer Unit (GE Healthcare, Piscataway, NJ). The quality of protein transfer was visually checked using pre-stained protein markers (Precision Plus Protein™ All Blue standards, Bio-Rad, Hercules, CA) and staining the membrane with Ponceau S (0.1% [w/v] in 5% [v/v] acetic acid). The membrane was blocked with 3% non-fat dry milk powder in 1X TBST buffer (10 mM Tris–HCl, Ph 8.0, 150 mM NaCl, 0.1% (v/v) Tween-20) for 1 h at room temperature with shaking.

Primary antibodies for immunoblots were as follows: RPS6 provided by Julia Bailey-Serres; BGAF provided by Asim Esen; GAPDH provided by Ming-Che Shih; eIF2α, eIF4E, and eIF4G provided by Karen Browning; SSIIa provided by Hanping Guan; SSIII, BEI and BEIIa/b provided by Alan Myers; anti-actin mouse monoclonal antibody (Cat. No. A0480, Sigma St. Lois MO). Membranes were incubated with primary antibodies diluted in TBST (1:1000 to 1:3000, based on the antibody titer) for 1 h at room temperature or overnight at 4°C, washed with TBST and then incubated for 1 h at room temperature with secondary antibodies (horseradish peroxidase-conjugated goat anti-rabbit or goat anti-mouse; Invitrogen, Carlsbad, CA) diluted in TBST (1:30,000). After washing with TBST, the membrane was treated with 1 ml SuperSignal West Pico Chemiluminescent Substrate (Pierce, Rockford, IL) for 2 min and the signals were detected using the Ultra- LUM Gel Imager System and UltraQuant 6.0 software (Ultra-Lum, Incorporated, Claremont, CA). The intensity of bands was quantified using the ImageJ software [70] and statistical differences of protein expression levels between W64A + and W64A*o2* were evaluated with unpaired two-tailed student’s t-test with the JMP statistical software (SAS Institute Inc., Cary, NC).

### 2D SDS-PAGE

Endosperms from mature kernels were isolated by soaking overnight in ddH_2_O at 4°C. Pericarp and embryo were removed and the endosperms dried in a freeze dryer; dried endosperms were ground to flour with a bead mill. Flour samples were extracted in borate extraction buffer with shaking overnight at 37°C [69]. Protein extracts were fractionated into zein and non-zein fractions by precipitation in 70% ethanol; the non-zein protein pellet was washed twice with 70% ethanol, dried and resuspended in IPG rehydration buffer (8 M urea, 2% CHAPS, 20 mM dithiothreitol, 0.005% bromophenol blue). Samples were loaded into immobilized pH 4–7 gradient strips directly during the rehydration of the gel. The first dimension separation was performed according to the manufacturer’s directions on either a Multiphor II or Ettan IPGphor 2 (GE Healthcare, Piscataway, NJ). The second dimension separation was performed using the Mini-Protein II vertical gel apparatus (Bio-Rad, Hercules, CA) according to the manufacturer’s directions. Proteins were visualized with Coomassie brilliant blue. Gels were compared and spot intensities quantified using Prodigy SameSpots gel analysis software (Nonlinear Dynamics, Newcastle upon Tyne, UK).

### Protein identification

Protein spots of interest were excised from the acrylamide gel and digested with trypsin using an in-gel digestion procedure. Briefly, gel pieces were destained by incubating in 50% acetonitrile 15 min at room temperature with two to three changes of solution. The gel pieces were dried in a Speed-Vac drier for 1 h, and were rehydrated by incubating in trypsin digestion buffer (50 mM ammonium bicarbonate, 5 mM CaCl_2_, 15 μg/ml sequencing grade trypsin (Sigma, St. Louis, MO)) for 10 minutes. Excess buffer was removed and enough trypsin-free digestion buffer was added to barely cover the gel pieces; the samples were then incubated overnight at 37°C. The buffer solution was removed to a fresh tube and the gel pieces were washed two times by incubating in 50% acetonitrile with 1% formic acid for 5 minutes and combined with the original supernatant. Peptides were concentrated and de-salted using C-18 Zip-Tips (Milipore, Billerica, MA) according to the manufacturer’s instructions.

Protein identification was performed by MALDI-TOF peptide mass mapping at the University of Arizona Mass Spectrometry facility. The peak lists derived from the mass spectra were searched against the Genbank non-redundant database updated 7/1/11 using the ProFound peptide mapping tool ([71]; http://prowl.rockefeller.edu/). Searches were performed using monoisotopic masses with the following parameters: taxonomy, other green plants; constant modification, iodoacetamide; Partial modification, methionine oxidation; mass tolerance, 200 ppm.

### Analysis of starch structure

Starch granules were purified essentially as described by Gutierrez *et al.*[72]. Mature kernels were soaked in 0.5% Na_2_S_2_O_5_ at 50°C for 24 h. The endosperm was dissected from the pericarp and germ, and ground lightly in a mortar. The sample was blended with 50 mM NaCl for 30 s and filtered through two layers of Miracloth (Calbiochem, San Diego, CA). The filtered material was extracted and pelleted by centrifugation five times in 1:4 toluene: 50 mM NaCl, followed by extraction two times with acetone. The starch was dried for 48 h before use.

Dried starch was weighed and suspended in a 1:3 [w:v] slurry in deionized water. The slurry was sealed in hermetic pans for DSC. The sample pan was loaded into the DSC instrument (Q200, TA Instruments, New Castle, DE) sample pedestal and the reference pedestal held an empty hermetic pan. The sample was equilibrated at 35°C and then a DSC scan was performed from 35–95°C heating at 5°C/min. The onset temperature, peak endotherm and total enthalpy were calculated using the TA Universal Analysis 2000 software (TA Instruments, New Castle, DE).

Amylopectin glucan chain length distributions were determined by capillary electrophoresis. Starch was dissolved in DMSO by heating to 95°C for 15 minutes. A small sample of the dissolved starch was debranched by isoamylase (Megazyme, Wicklow, Ireland) and the resulting glucans were labeled with 8-amino-1,3,6-pyrenetrisulfonic acid in the presence of sodium cyanoborohidride. A sample of the labeling reaction was diluted and the fluroescently labeled glucan chains were separated and quantified by fluorescence-assisted capillary electrophoresis. Histograms of the percentage of area for each peak were plotted and compared.

## Abbreviations

AAA-ATPase: Proteosome regulatory subunit AAA-ATPase; AFLP: Amplified fragment length polymorphism; AGP: Arabinogalactan protein; BAP2: Basal layer antifungal protein2; BEI: Starch branching enzyme I; BEIIa: Starch branching enzyme IIa; BEIIb: Starch branching enzyme IIb; BGAF: Beta-glucosidase aggregating factor; CI-1B: Subtilisin-chymotrypsin inhibitor CI-1B; DSC: Differential scanning calorimetry; DAP: Days after pollination; EF1A: Translation elongation factor 1 alpha; eIF2α: Eukaryotic translation initiation factor 2 alpha subunit; eIF4E: Eukaryotic translation initiation factor 4E; eIF4G: Eukaryotic translation initiation factor 4G; ER: Endoplasmic reticulum; ERO1: ER Membrane-Localized Oxidoreductase 1; GAPDH: glyceraldehyde-3-posphate dehydrogenase; N4: General control non-derepressible 4; GDSL: Glycine–aspartic acid–serine-leucine; GBSSI: Granule-bound starch synthase I; HSP3: 17.4-kDa Class I heat shock protein 3; IF5A: Translation initiation factor 5A; LKR-SDH1: Lysine-ketoglutarate reductase/saccharopine dehydrogenase1; MAPK: Mitogen-activated protein kinase; MEE21: Maternal effect embryo arrest 21; NAC: NAM, ATAF, and CUC transcription factor; o2: Opaque2; PDI: Protein disulfide isomerase; PGK: Cytosolic phosphoglycerate kinase; qRT-PCR: Quantitative real-time polymerase chain reaction; RBOHB: Respiratory burst oxidase protein homolog B; RIP: Ribosome-inactivating protein; RNAi: RNA interference; ROS: Reactive oxygen species; RRB1: Retinoblastoma-related protein 1; S6RP: Ribosomal protein S6; SDH1: Sorbitol dehydrogenase 1; SSIIa: Starch synthase IIa; SSIII: Starch synthase III; TSJT1: Stem-specific protein; TA1: Tryptophan aminotransferase; TIM: Triose phosphate isomerase; Zpu1: Pullulanase-type starch debranching enzyme1.

## Competing interests

The authors declare no competing interests.

## Authors’ contributions

MJ Experimental data and writing. KC Experimental data. HW Experimental data. RJ Experimental design and data. BAL Writing and experimental design. BCG Writing, experimental design and data. All authors read and approved the final manuscript.

## Supplementary Material

Additional file 1: Table S1 Gene expression values for differentially expressed bands with confirmed sequences. Click here for file

Additional file 2: Figure S1 Correlation analysis of qRT-PCR and transcript profiling gene expression values. To examine reproducibility for measurement of gene expression, the values for genes confirmed by qRT-PCR were plotted against the values measured by GeneCalling transcript profiling. A Pearson correlation analysis was performed (r = 0.80) and the statistical significance of the linear regression was tested by ANOVA (p < 0.001). The value of b-32 from qRT-PCR was determined as a significant outlier by Grubbs’ test and therefore the fold-change values of b-32 from both tests were removed from the plot and regression analysis. Click here for file

Additional file 3 **Sequence tags used to determine gene identities.** FastA formatted sequence text file. The sequences for each band that were confirmed by either competitive PCR or sequencing of the band were used to determine gene identitiy by searching the non-redundant genbank database using the basic local alignment and search tools BLASTN and BLASTX. Click here for file

Additional file 4: Table S2 qRT-PCR primer sequences. Click here for file
