# Supplementary material for: Identification and characterization of lysine-rich proteins and starch biosynthesis genes in the opaque2 mutant by transcriptional and proteomic analysis
Source: BMC Plant Biol. 2013 Apr 12;13:60. doi: 10.1186/1471-2229-13-60 (PMC3762070; doi:10.1186/1471-2229-13-60)
Supplement: Additional file 1: Table S1 — Gene expression values for differentially expressed bands with confirmed sequences. [file 1471-2229-13-60-S1.docx]

Additional Table 1. Gene expression values for differentially expressed bands with confirmed sequences.

| Band ID | Identity/Top BLAST hit | Relative expression^1^ | GO Biological Process |
| --- | --- | --- | --- |
| l0r0-176.5 | GDSL-motif lipase/hydrolase-like protein [Zea mays] | -42.94 | oil metabolism |
| r0k0-378.2 | Putative retrotransposon protein [Zea mays] | -7.57 | carbohydrate metabolism |
| u0w0-144.3 | Unknown | -6.65 | unknown |
| u0w0-247.4 | Triosephosphate isomerase, cytosolic [Zea mays] | -5.46 | carbohydrate metabolism |
| m1l0-250.7 | Phenylcoumaran benzylic ether reductase -like protein [Populus trichocarpa] | -5.25 | lignan biosynthesis |
| m1a0-73.6 | 19kD β-zein | -4.71 | nutrition reservoir |
| s0w0-342.8 | Phosphoglycerate kinase, cytosolic [Zea mays] | -4.02 | carbohydrate metabolism |
| l0n0-46.1 | Unknown | -4.02 | Unknown |
| m1g1-126.6 | Mannose-1-phosphate guanyltransferase [Zea mays] | -3.83 | carbohydrate metabolism |
| u0f0-75.4 | 16kD zein | -3.7 | nutrition reservoir |
| u0w0-263.8 | Aspartic proteinase oryzasin-1 [Zea mays] | -3.33 | protein turnover |
| i0c0-41.5 | Coatomer subunit gamma | -2.96 | Transport |
| m0v0-122.7 | Cystatin6 [Zea mays] | -2.9 | defense response |
| m1e1-262.5 | Alanine aminotransferase 2 [Zea mays] | -2.87 | amino acid metabolism |
| s0w0-364.5 | Alcohol dehydrogenase 1 (ADH1) | -2.86 | carbohydrate metabolism |
| m1a0-180.7 | 22kD zein | -2.68 | nutrition reservoir |
| d0g0-203.9 | Nuclear protein [Zea mays] | -2.61 | Unknown |
| u0w0-166.5 | DNA-directed RNA polymerases I, II, and III 17.1 kDa polypeptide [Zea mays] | -2.55 | transcription/translation |
| d0g0-241.4 | Farnesyl pyrophosphate synthase [Zea mays] | -2.45 | lipid metabolism |
| g1n0-446.0 | Rhodopsin-like receptor [Zea mays] | -2.39 | signal transduction |
| u0v0-293.2 | CHITINASE B | -2.38 | carbohydrate metabolism |
| i0l0-390.0 | Protein disulfide isomerase [Zea mays] | -2.37 | protein folding |
| u0g1-218.4 | Putative VP2-like RING Finger TF \| VIP2 protein [Avena fatua] | -2.35 | transcription/translation |
| r0k0-193.9 | Pullulanase-type starch debranching enzyme1 [Zea mays] | -2.27 | carbohydrate metabolism |
| g1n0-261.1 | 2PGK 2-phosphoglycerate kinase [Arabidopsis thaliana] | -2.25 | carbohydrate metabolism |
| i0r0-82.0 | 16.9 kDa class I heat shock protein 3 [Zea mays] | -2.22 | stress response/defense response |
| g0m0-51.6 | ZIP zinc/iron transport family protein [Zea mays] | -2.21 | Transport |
| l0r0-89.8 | Pol protein homolog - maize retrotransposon | -2.19 | Unknown |
| i0n0-159.3 | Oryza sativa GDP dissociation inhibitor protein OsGDI1 | -2.16 | signal transduction |
| l0r0-186.0 | Respiratory burst oxidase protein B [Zea mays] | -2.15 | oxidation-reduction process/defense response |
| u0e1-199.0 | Sorbitol dehydrogenase homolog1 [Zea mays] | -2.09 | carbohydrate metabolism |
| i0n0-200.4 | Calnexin [Zea mays] | -2.08 | protein folding |
| m1a0-110.4 | Actin | -1.99 | Cytoskeleton |
| g1n0-284.0 | WRKY17 transcription factor [Triticum aestivum] | -1.97 | transcription/translation |
| l0r0-318.4 | Unknown | -1.91 | Unknown |
| u0g1-173.2 | MAP kinase [Zea mays] | -1.9 | signal transduction |
| d0g0-384.4 | Epoxide hydrolase 2 [Zea mays] | -1.87 | catalytic activity |
| l0u0-78.0 | Fructokinase-1 [Zea mays] | -1.85 | carbohydrate metabolism |
| i0l0-124.9 | Granule-bound starch synthase 1 [Zea mays] | -1.84 | carbohydrate metabolism |
| u0g1-418.8 | Calreticulin2 [Zea mays] | -1.83 | protein folding |
| i0r0-116.8 | NAM-related protein 1 [Zea mays] | -1.83 | transcription/translation |
| m1w0-162.3 | 19KD zein | -1.81 | nutrition reservoir |
| u0e1-53.0 | Pi starvation-induced protein [Zea mays]. | -1.8 | defense response |
| l0n0-236.7 | Unknown | -1.8 | Unknown |
| m1l0-408.1 | Mitochondrial 2-oxoglutarate/malate carrier protein [Zea mays] | -1.78 | Transport |
| l0n0-122.1 | RING-H2 finger protein ATL1R [Zea mays] | -1.76 | transcription/translation |
| l0u0-281.0 | Grancalcin [Zea mays] | -1.75 | signal transduction |
| u0g1-84.4 | 60S ribosomal protein L33-B [Zea mays] | -1.75 | transcription/translation |
| d0g0-209.4 | 1,4-alpha-glucan-branching enzyme 2, chloroplastic/amyloplastic precursor [Zea mays] | -1.74 | carbohydrate metabolism |
| m1n0-168.8 | Protein transport protein SEC31 [Arabidopsis thaliana] | -1.74 | Transport |
| u0e1-278.1 | Globulin precursor [Zea mays] | -1.73 | nutrition reservoir |
| i0n0-209.1 | Protein kinase-like domain containing protein [Oryza sativa] | -1.7 | signal transduction |
| u0v0-41.2 | Legumin 1 [Zea mays] | -1.69 | nutrition reservoir |
| r0s0-183.7 | Ethylene receptor homolog2 [Zea mays] | -1.67 | signal transduction |
| m1a0-228.5 | 27kD gamma zein | -1.66 | nutrition reservoir |
| r0a0-209.1 | Osr40g2 [Oryza sativa] | -1.63 | defense response |
| m1w0-94.5 | Zea mays heat shock protein18c (hsp18c) | -1.63 | defense response |
| m1a0-153.9 | Chaperone DNA J2 [Zea mays] | -1.61 | protein folding |
| l0m0-197.2 | Xylanase inhibitor protein 1 [Zea mays] | -1.59 | defense response |
| g1n0-213.8 | F-box protein FBL2 [Zea mays] | -1.57 | protein turnover |
| l0e1-164.1 | Unknown | -1.57 | Unknown |
| m1e1-92.0 | Alliin lyase 2 [Zea mays] | -1.53 | defense response |
| l0n0-82.6 | Trehalose 6 phosphate synthase | -1.52 | secondary metabolism |
| m0r0-95.0 | Unknown | -1.52 | Unknown |
| f0i0-327.7 | Symplekin [Arabidopsis thaliana] | -1.5 | transcription/translation |
| d0l0-411.9 | Vacuolar sorting receptor 1 precursor [Zea mays] | -1.5 | Transport |
| i0r0-186.8 | ERO1 | -1.48 | oxidation reduction |
| u0e1-147.7 | Alpha globulin [Zea mays] | -1.39 | nutrition reservoir |
| i0l0-368.8 | set1 complex component swd2 [Zea mays] | -1.35 | signal transduction |
| l0r0-240.1 | RNA binding protein Rp120 [Oryza sativa Japonica Group] | -1.33 | transcription/translation |
| i0u0-149.5 | 17.4 kDa class I heat shock protein 3 [Zea mays] | 1.16 | defense response |
| r0a0-136.7 | Acyl-CoA binding protein [Zea mays] | 1.54 | oil metabolism |
| l0r0-126.8 | Unknown | 1.54 | Unknown |
| i0a0-180.4 | 40S ribosomal protein S3a [Zea mays] | 1.6 | transcription/translation |
| u0e1-281.6 | 27kD gamma zein | 1.62 | nutrition reservoir |
| m1a0-254.1 | 22kD zein | 1.62 | nutrition reservoir |
| i0u0-342.7 | Unknown | 1.68 | Unknown |
| n0s0-143.3 | Gamma-interferon-inducible lysosomal thiol reductase [Zea mays] | 1.7 | catalytic activity |
| l0n0-252.8 | 60S ribosomal protein L19-3 [Zea mays] | 1.72 | transcription/translation |
| i0a0-267.0 | Unknown | 1.72 | Unknown |
| r0a0-306.9 | 27kD gamma zein | 1.74 | nutrition reservoir |
| i0q0-74.7 | Omega-6 fatty acid desaturase, endoplasmic reticulum isozyme 2 [Zea mays] | 1.74 | oil metabolism |
| n0s0-44.1 | Brassinosteroid biosynthesis-like protein [Zea mays] | 1.8 | sterol metabolism |
| u0g1-105.5 | 19kD D zein | 1.81 | nutrition reservoir |
| d0l0-156.0 | Ketol-acid reductoisomerase [Zea mays] | 1.85 | amino acid metabolism |
| m1g1-303.2 | 19kD D zein | 1.9 | nutrition reservoir |
| u0v0-116.1 | 27kD gamma zein | 1.94 | nutrition reservoir |
| n0s0-200.4 | Chorismate mutase [Zea mays] | 1.94 | secondary metabolism |
| i0u0-181.8 | Basal layer antifungal protein2 [Zea mays] | 1.94 | defense response |
| m1n0-361.7 | Pyruvate dehydrogenase E1 alpha subunit [Zea mays] | 1.95 | carbohydrate metabolism |
| u0e1-267.8 | Oleosin 18 kDa [Zea mays] | 1.96 | nutrition reservoir |
| i0l0-411.0 | Dehydration-responsive protein RD22 [Prunus persica] | 2.01 | defense response |
| m1e1-112.2 | 19KD zein | 2.07 | nutrition reservoir |
| w0c0-127.0 | ERF-like protein [Zea mays] | 2.07 | transcription/translation |
| m1l0-86.4 | 18S ribosomal RNA gene | 2.07 | transcription/translation |
| i0a0-145.9 | Miniature seed1 [Zea mays] | 2.18 | carbohydrate metabolism |
| u0e1-260.3 | 27kD gamma zein | 2.19 | nutrition reservoir |
| d0v0-156.9 | 27kD gamma zein | 2.2 | nutrition reservoir |
| w0c0-71.6 | Probable non-specific lipid-transfer protein 2 (LTP 2) [Zea mays] | 2.22 | Transport |
| n0s0-146.7 | NAC domain-containing protein 48 [Zea mays] | 2.28 | transcription/translation |
| d0v0-87.8 | Katanin p60 ATPase-containing subunit [Zea mays] | 2.41 | Cytoskeleton |
| i0u0-49.4 | Flavin monooxygenase [Zea mays] | 2.41 | secondary metabolism |
| i0q0-71.9 | Alanine--glyoxylate aminotransferase 2 [Zea mays] | 2.44 | amino acid metabolism |
| f0i0-181.3 | 19kD D zein | 2.46 | nutrition reservoir |
| s0w0-228.1 | 19KD zein | 2.51 | nutrition reservoir |
| m1a0-183.7 | 22kD zein | 2.52 | nutrition reservoir |
| l0n0-327.7 | Heat shock protein 90 [Zea mays] | 2.53 | defense response |
| m1s0-396.5 | 19KD zein | 2.57 | nutrition reservoir |
| u0e1-209.6 | 27kD gamma zein | 2.7 | nutrition reservoir |
| y0i0-433.7 | Alanine--glyoxylate aminotransferase 2 [Zea mays] | 2.72 | amino acid metabolism |
| h0a0-346.2 | Glutathione S-transferase GST 31 [Zea mays] | 2.79 | signal transduction |
| f0i0-156.8 | Unknown | 2.85 | Unknown |
| m1l0-273.4 | PIN domain-containing MEE21 protein [Arabidopsis thaliana] | 2.9 | embryo development |
| i0u0-440.7 | 22kD zein | 2.95 | nutrition reservoir |
| t0w0-138.0 | Zein-alpha 19C2 precursor [Zea mays] | 2.96 | nutrition reservoir |
| d0g0-177.0 | 19kD B zein | 3.11 | nutrition reservoir |
| d0g0-191.0 | 19kD D zein | 3.13 | nutrition reservoir |
| n0s0-191.7 | Zea mays protein b-32 | 3.27 | defense response |
| u0v0-169.7 | Aquaporin TIP3.1 [Zea mays] | 3.42 | Transport |
| y0i0-281.4 | Plastidic phosphate translocator-like protein1 [Zea mays] | 3.46 | Transport |
| i0n0-434.4 | Arabinogalactan protein [Zea mays] | 3.49 | Cell wall |
| f0i0-309.9 | Protein FAR-RED IMPAIRED RESPONSE 1 [Arabidopsis thaliana | 3.58 | signal transduction |
| h0a0-79.7 | Plastid phosphate/phosphoenolpyruvate translocator1 [Zea mays] | 3.72 | Transport |
| u0e1-45.3 | Flower-specific gamma-thionin [Zea mays] | 3.75 | defense response |
| n0s0-298.4 | Unknown | 3.79 | Unknown |
| i0q0-229.3 | Flower-specific gamma-thionin [Zea mays] | 3.85 | defense response |
| y0i0-156.3 | 22kD alpha zein4 | 3.99 | nutrition reservoir |
| f0i0-79.9 | YT521-B-like family protein, expressed [Oryza sativa Japonica Group] | 4.07 | signal transduction |
| m1e1-299.9 | 22kD zein | 4.42 | nutrition reservoir |
| s0w0-316.7 | 22kD zein | 4.95 | nutrition reservoir |
| u0g1-56.3 | 18kD delta zein | 5.05 | nutrition reservoir |
| m0v0-207.8 | AAA-type ATPase family protein [Arabidopsis lyrata subsp. lyrata] | 5.27 | energy metabolism |
| m1n0-181.0 | 22kD zein | 5.4 | nutrition reservoir |
| l0m0-392.4 | Opie2 pol protein [Zea mays] | 5.5 | DNA integration |
| n0s0-287.1 | Stem-specific protein TSJT1 [Zea mays] | 5.61 | protein turnover |
| h0a0-42.1 | Oleosin Zm-I [Zea mays] (Oleosin 16kda) | 5.7 | nutrition reservoir |
| m1e1-372.6 | 19KD zein | 6.86 | nutrition reservoir |
| l0n0-375.0 | Beta-glucosidase aggregating factor [Zea mays] | 7.64 | defense response |
| u0f0-432.4 | 22kD zein | 8.37 | nutrition reservoir |
| r0k0-386.9 | Subtilisin-chymotrypsin inhibitor CI-1B [Zea mays] | 9.4 | defense response |
| s0w0-394.9 | 22kD zein | 9.56 | nutrition reservoir |
| m1w0-272.1 | 19KD zein | 10.37 | nutrition reservoir |
| u0e1-433.0 | Zea mays protein b-32 | 10.67 | defense response |
| u0g1-426.3 | 22kD zein | 17.46 | nutrition reservoir |
| b0w0-179.8 | 22kD zein | 20.48 | nutrition reservoir |
| m1s0-153.1 | 22kD zein | 25.74 | nutrition reservoir |
| s0w0-226.0 | 22KD zein | 30.02 | nutrition reservoir |
| m1w0-350.7 | 22kD zein | 65.11 | nutrition reservoir |
| u0e1-68.2 | 22kD zein | 70.61 | nutrition reservoir |
| m1e1-226.8 | 22kD zein | 81.88 | nutrition reservoir |
| m1a0-88.3 | Tryptophan aminotransferase (TA1) [zea mays] | 100 | amino acid metabolism |

^1^Expression values are fold-change. Negative values indicate increased expression in W64A*o2* relative to W64A wild type.
