# Supplementary figures and images for: Identification and characterization of lysine-rich proteins and starch biosynthesis genes in the opaque2 mutant by transcriptional and proteomic analysis
Source: BMC Plant Biol. 2013 Apr 12;13:60. doi: 10.1186/1471-2229-13-60 (PMC3762070; doi:10.1186/1471-2229-13-60)

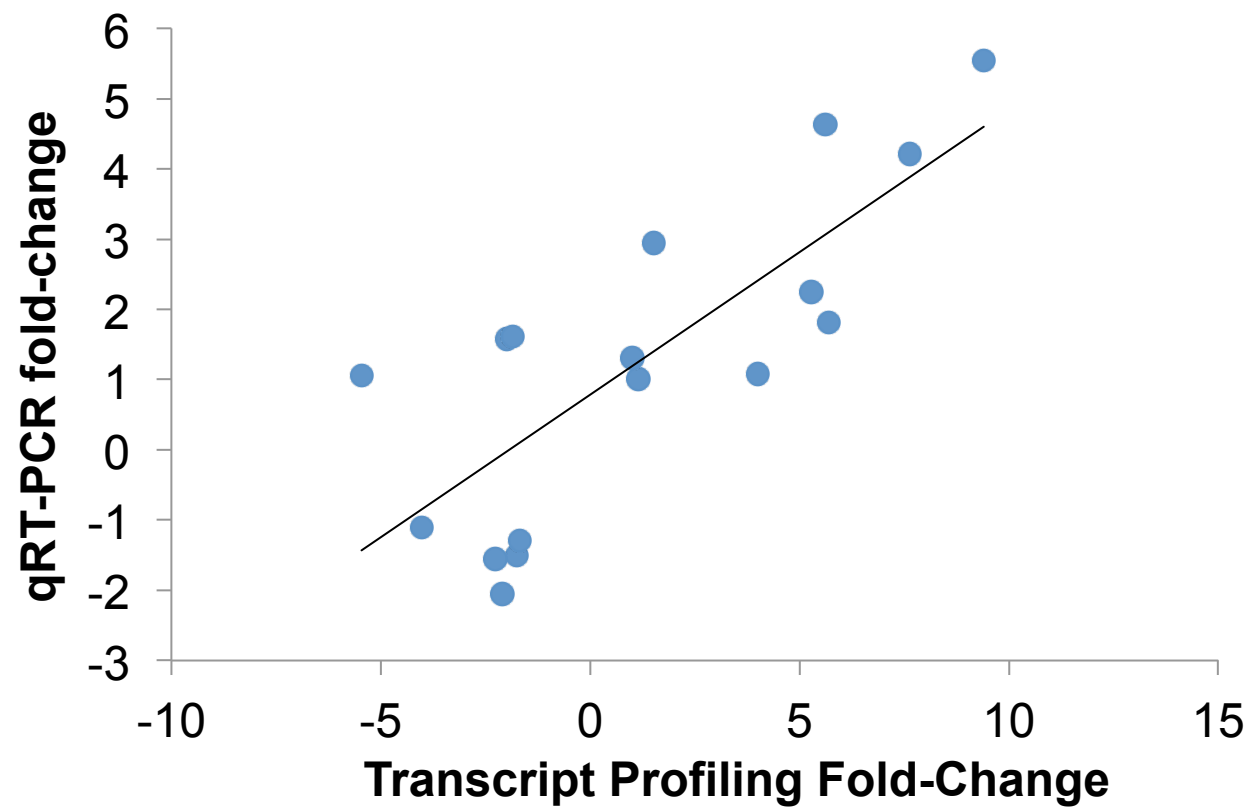

Supplement: Additional file 2: Figure S1 — Correlation analysis of qRT-PCR and transcript profiling gene expression values. To examine reproducibility for measurement of gene expression, the values for genes confirmed by qRT-PCR were plotted against the values measured by GeneCalling transcript profiling. A Pearson correlation analysis was performed (r = 0.80) and the statistical significance of the linear regression was tested by ANOVA (p < 0.001). The value of b-32 from qRT-PCR was determined as a significant outlier by Grubbs’ test and therefore the fold-change values of b-32 from both tests were removed from the plot and regression analysis. [file 1471-2229-13-60-S2.pdf]
